# Function verification of a chlorophyll a/b binding protein gene through a newly established tobacco rattle virus-induced gene silencing system in *Kandelia obovata*

**DOI:** 10.3389/fpls.2023.1245555

**Published:** 2023-10-03

**Authors:** Mingxiong Zhang, Yuhui Rao, Xiaofeng Chen, Yunrui Shi, Chonglong Wei, Xianfeng Wang, Lu Wang, Chengjin Xie, Chenglang Pan, Jianming Chen

**Affiliations:** ^1^ Fujian Key Laboratory on Conservation and Sustainable Utilization of Marine Biodiversity, Fuzhou Institute of Oceanography, Minjiang University, Fuzhou, China; ^2^ College of Resource and Environmental Science, Fujian Agriculture and Forestry University, Fuzhou, China; ^3^ Technology Innovation Center for Monitoringand Restoration Engineering of Ecological Fragile Zonein Southeast China, Ministry of Natural Resources, Fuzhou, China; ^4^ College of Environment and Safety Engineering, Fuzhou University, Fuzhou, China

**Keywords:** *Kandelia obovata*, VIGS system, phytoene desaturase, chlorophyll a/b binding protein, carbon sequestration

## Abstract

As an important mangrove species, *Kandelia obovata* plays an irreplaceable role in the coastal ecosystem. However, due to a lack of genetic technology, there is limited research on its functional genes. As such, establishing an efficient and rapid functional verification system is particularly important. In this study,tobacco rattle virus (TRV) and the phytoene desaturase gene *KoPDS* were used as the vector and target gene, respectively, to establish a virus-induced gene silencing system (VIGS) in *K. obovata*. Besides, the system was also used to verify the role of a Chlorophyll a/b binding protein (Cab) gene *KoCAB* in leaf carbon sequestration of *K. obovata.*

RNA-Seq and qRT-PCR showed that the highest gene-silencing efficiency could reach 90% after 10 days of inoculation and maintain above 80% after 15 days, which was achieved with resuspension buffer at pH 5.8 and *Agrobacterium* culture at OD_600_ of 0.4-0.6. Taken together, the TRV-mediated VIGS system established herein is the first genetic analysis tool for mangroves, which may greatly impel functional genomics studies in mangrove plants.

## Introduction

*Kandelia obovata* is the dominant mangrove species along the southeast coast of China and is tolerant to a variety of complex environments such as high organic matter, low oxygen, and tidal flooding in the sea-land ecotone (Senger et al., 2021; Jiang et al., 2022). In addition, it is an integral part of blue carbon ecosystems, which can absorb large quantities of carbon dioxide from the atmosphere and alleviate the negative impact of global climate change. Li LF. et al. (2015) showed that the daily net carbon sequestration of *K.obovata* was 13.83 g·m^-2^·d^-1^ in the Zhanjiang mangrove forest area, which was significantly more than that of *Avicennia marina*, *Aegiceras corniculatum*, *Bruguiera gymnorrhiza*, and *Rhizophora stylosa*. Traditional studies have shown that a wide range of factors affect the photosynthetic efficiency of *K.obovata*; however, the primary regulatory mechanism remains unclear.

The chlorophyll a/b binding proteins (Cab) are a class of membrane proteins that bind to pigment molecules in the plant photosystem. They are encoded by nuclear genes and are widely involved in light energy capture, energy transfer, and respond to various stresses. Previous studies revealed that *K.obovata* chlorophyll a/b binding protein *KoCab* (OR479794) is significantly differentially expressed during photosynthesis, but its exact function is still unknown. Analysis of the functions of *KoCab* underlying photosynthetic carbon sequestration is critical for enhancing our understanding of the special physiological and ecological characteristics of mangrove plants. It can also provide guidance for further improving the carbon sequestration efficiency of mangrove plants during mangrove restoration.

Although it is currently possible to characterize the function of mangrove genes, to some extent, using the model plants such as *Arabidopsis thaliana* (Hong et al., 2018) and rice (Prashanth et al., 2008), results from heterologous transformations may not faithfully reveal the biological function of the target gene in mangrove plants. Moreover, *K. obovata* is rich in secondary metabolites such as tannin, which can cause leaf hypersensitivity when transformation systems of common model plants are used. As such, researches on mangrove plants at the molecular level lag far behind those of other model plants.

Virus-induced gene silencing (VIGS) is a powerful reverse genetics technology that can be used for rapid analysis of gene function. To verify the function of target genes, VIGS uses virus vectors to carry cDNA fragments from target genes to induce gene silence and related phenotypic mutation in plant tissues (Li et al., 2021). Virus vectors currently used for VIGS mainly include DNA virus vectors, RNA virus vectors, and satellite virus vectors. Tobacco rattle virus (TRV) is a soil-borne RNA virus and belongs to the genus Tobravirus (Senthil-Kumar and Mysore, 2014). Due to the wide host range, high silencing efficiency, and mild virus symptoms associated with rapid propagation in plant meristems, TRV has become the preferred vector for most VIGS systems (Sui et al., 2018). At present, many studies have reported the use of TRV-VIGS technology in gene function research for many non-model plants, such as the carotenoid cleavage dioxygenase gene of peach (Bai et al., 2015), the phytoene desaturase (*PDS*) gene of strawberry (Tian et al., 2015), the chalcone isomerase gene of apple (Wang, 2014), the P450 monooxygenase gene of cherry (Li Q. et al., 2015), the flavonoid-3-O-glucosyltransferase gene of *Litchi chinensis* (Li X. et al., 2015), the acyltransferase 3 gene *AT3* of *Capsicum annuum* (Arce-Rodríguez et al., 2015), the actin depolymerizing factor gene *TaADF7 of* wheat (Chen et al., 2022), the neomycin phosphotransferase gene in cotton (Wu et al., 2021), the methionine sulfoxide reductase gene of eggplant (Zhao et al., 2015), and *PDS* in the beet genome (Gong et al., 2015). However, whether TRV-VIGS technology is suitable for mangrove plants is unclear, as is how a TRV-VIGS silencing system for mangrove plants should be constructed.

The phytoene desaturase gene is a rate-limiting enzyme involved in carotenoid synthesis in plants, and *PDS* silencing can generate obvious chlorosis of green plant tissues which is easy for detection (Zeng et al., 2019). Therefore, in this study, the TRV virus and the *K. obovata* phytoene desaturase gene *KoPDS* were used respectively as the virus vector and the marker gene to establish a VIGS system in *K. obovata*. We also used the established system, together with a comparative transcriptomics analysis, to verify the function of the *Cab* gene in *K. obovata*. The establishment of the VIGS system in *K. obovata* can promote functional verification of genes that are related to important traits of mangroves and provide technical support for later high-throughput functional genomics analysis.

## Materials and methods

### Plant materials

Mature *K. obovata* propagules were collected from the Zhangjiang estuary in Fujian Province, China (23°53′45″-23°56′00″N, 117°24′07″-117°30′00″E). The propagules had been cultivated in pots containing sand and irrigated with Hoagland’s nutrient solution _for_ one year. The plants were then placed in a greenhouse under controlled conditions [1000 µmol photons m^-2^s^-1^, 28/25°C (day/night)] for cultivation and experiments. Uniform size robust seedlings (1-year cultivation) were selected and the 3rd–5th mature leaves from the top were used for studies.

### Vector construction

The extraction of total RNA from the samples was carried out based on the manufacturer’s instructions for the Easy Spin plus complex plant RNA kit. cDNA synthesis was performed using a PrimeScript RT reagent kit with gDNA Eraser (Takara, Dalian, China). The gene fragments (*KoPDS and KoCab*) were amplified using specific primers (*KoPDS*-OF, *KoPDS*-OR, *KoCab*-OF, and *KoCab*-OR) and inserted into pTRV2 to generate pTRV2-*KoPDS and* pTRV2-*KoCab*, respectively. The primer sequences used for vector construction are listed in **Supplementary Table 1**.

### RNAi transient assay using a virus-induced gene silencing system

After the vector sequence was verified by sequencing, pTRV1, pTRV2, and pTRV2-*KoPDS* were individually transformed into the *Agrobacterium* strain GV3101. Seeding solutions were prepared by overnight incubation of the transformed *Agrobacterium* in LB medium containing kanamycin (Kan) and rifampicin (Rif) at 28°C with shaking at 250 rpm. The solutions were then diluted in fresh LB (Kan + Rif) medium at a ratio of 1:50 (vol:vol) and were then placed on an incubator shaker and grown to the required concentration as measured by OD_600_ and then centrifuged at 4,000 rpm for 10 min. Then, pTRV1 and pTRV2-*KoPDS* (pTRV1 and pTRV2 as a control) were mixed with the same volume of infection buffer, and the infection mixture was allowed to stand for 2-4 hours at 28°C before injection into plants. Infection buffer solution (0.1 mM MES, 0.1 mM MgCl_2_·6H_2_O, with 200 µM acetosyringone in 100 mL dd H_2_O) was adjusted to the indicated pH value (pH 4.5-6.5) with 1M NaOH. For infection, the topmost pair of mature leaves were pierced with a syringe needle and the indicated mixture (pTRV1/pTRV2-*KoPDS*; pTRV1/pTRV2 as a control) was injected into 1-2 blades of leaves under the meristem via the wound, so that the mixture could completely infect the entire leaf. The leaves used for the three replicates were selected from different mangrove plants, such that no replicates for one experimental condition were taken from the same plant. Phenotype silencing in the plants appeared within 7-15 days after incubation at room temperature (lower than 28°C) with protection from light.

### Evaluation of infection effect

To evaluation the infection effect of the TRV, we used the total chlorophyll content of the leaves and effective infection rate as the reference indicator. Chlorophyll was quantitatively determined with the Meter Model SPAD-502 (SPAD). The effective infection rate is the ratio of the leaves with chlorosis symptoms near the infection point to the total number of leaves receiving the same treatment. Based on the results of the previous experiments, the total chlorophyll content and effective infection rate were determined starting at 7 days after infection.

### RT-qPCR analysis of *KoPDS* expression levels

*K. obovata* leaves infected with TRV for 7, 10, and 15 days were sampled and pooled into three replicates that were immediately frozen in liquid nitrogen and kept at -80°C for use in further experiments. RNA extraction and reverse transcription were performed as described above. qRT-PCR was performed using the Eppendorf Realplex 4 real-time PCR system (Hamburg, Germany) as described previously (Fang et al., 2016). *KoPDS*-F/R was designed to isolate and clone the *KoPDS* coding region of the phytoene desaturase gene from *K. obovata*. The *Koactin* gene was used as a reference. Expression levels were calculated as described previously (Fang et al., 2016).

### Comparative transcriptome to verify the function of the *Cab* gene in *K. obovata*

*K. obovata* leaves infected with the pTRV1+pTRV2 vector or pTRV1+pTRV-*KoCab* (described above) for 15 days were sampled and were used for transcriptome analysis. Extraction of total RNA, library construction, and RNA-Seq were performed by staff at Beijing BioMarker Technologies (Beijing, China). Sequencing of the cDNA library was carried out on the Illumina HiSeqTM X Ten sequencing platform. RNA-seq was performed in three replicates (Fang et al., 2021). Photosynthetic parameters were measured in an incubation chamber at 25°C with a light intensity of 400 μmol·m^-2^·s^-1^. *KoCab* expression levels were analyzed by RT-qPCR as described above.

## Results

### Construction of the TRV-VIGS system

**Figure 1:**
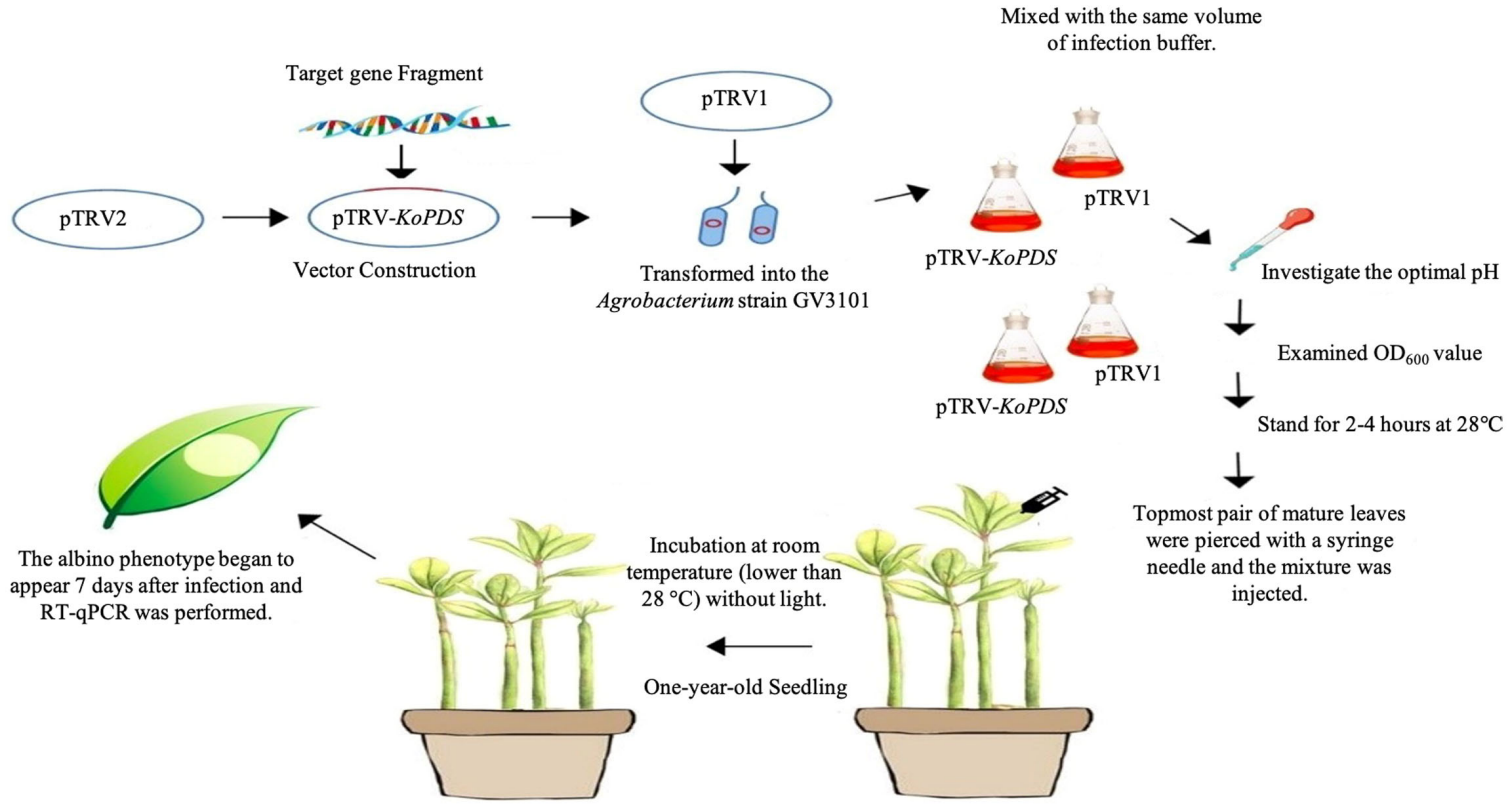
Overview of the VIGS protocol in *K. obovata*.

An overview of the protocol for *Agrobacterium*-mediated delivery of TRV1 and TRV2 constructs into *K. obovata* by syringe inoculation was shown in **Figure 1**. By using TRV-VIGS, *KoPDS* can be silenced in 7 days after TRV inoculation and used for subsequent analysis. In this study, the pTRV2 vector was digested with *EcoRI* and *KpnI*, and the *KoPDS* fragment (312 bp) was used to construct the recombinant pTRV2-*KoPDS* vector (**Supplementary Data Figure S1**).

**Figure 2:**
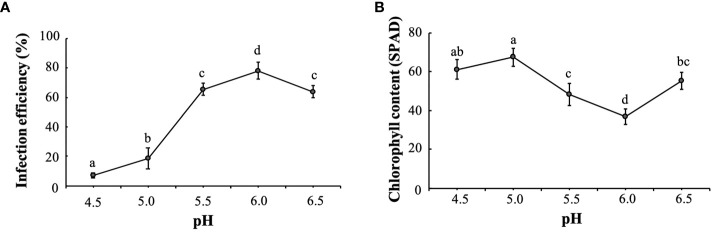
Optimal infection pH for the *K. obovata* VIGS system. **(A)** Effect of resuspension buffers having different pH on infection efficiency. **(B)** Effect of resuspension buffers having different pH on leaf chlorophyll content. Statistical analyses were performed using SPSS 17.0, and significance was assessed with Tukey’s Test at P < 0.05. All experiments included three biological replicates. Five leaves derived from five individual plants were collected for each biological replicate in each experiment. Different letters indicate that the data are directly significantly different (P < 0.05).

Chlorosis symptoms appeared on the leaves about 7 days after infection, followed by a gradual whitening of the spots that did not further intensify after about 15 days. As shown in **Figure 2A**, the infection rate of *K. obovata* leaves was significantly and positively correlated with pH at 5.0-6.0, and the infection efficiency was maximized at pH 6.0. The total chlorophyll content was negatively correlated with pH values. As the pH increased from 5.0 to 6.0, the chlorophyll content decreased significantly and reached its lowest value at pH 6.0. When the pH value exceeded 6.0 to 6.5, the infection efficiency declined, and the leaves wilted to different degrees (**Figure 2B**). Therefore, the data show that the optimum re-suspension pH of the VIGS system is 6.0, which is where the optimum VIGS infection efficiency was achieved.

**Figure 3:**
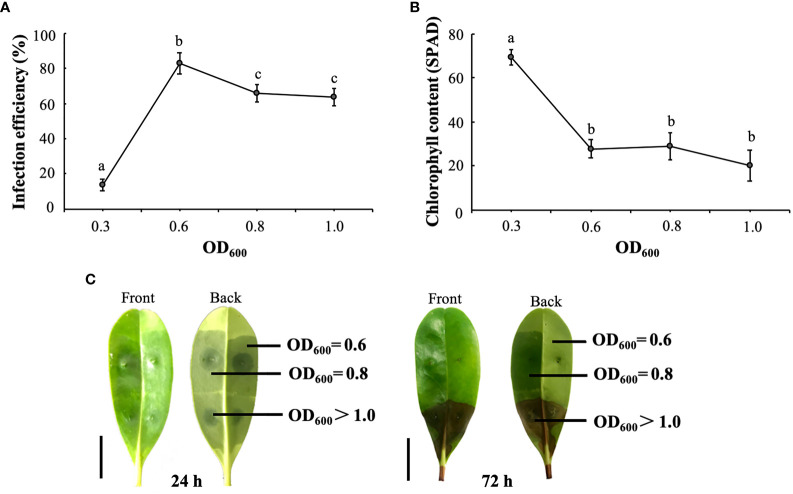
Optimal OD_600_ of the *K. obovata* VIGS system. **(A)** Effect of different OD_600_ values on infection efficiency. **(B)** Effect of different OD_600_ on leaf chlorophyll content. **(C)** Albino phenotype of leaves 24 and 72 hours after infection. The front and back of the leaf were showed, respectively. At 72 hours after infection, necrotic spots began to appear at OD_600_ >1. The scale bar on the left corresponds to 1 cm. Statistical analyses were performed using SPSS 17.0, and significance was assessed with Tukey’s Test at P < 0.05. All experiments included three biological replicates. Five leaves derived from five individual plants were collected for each biological replicate in each experiment. Different letters indicate that the data are directly significantly different (P < 0.05).

Based on the results, equal volumes of pTRV1 and pTRV2-*KoPDS* were injected into a resuspension solution at pH 6.0 and then into the abaxial surface of leaves. Chlorosis symptoms began to appear approximately 7 days after incubation. The results of the infection efficiency showed that the increase in the agrobacterium density (OD_600_) was significantly and positively correlated with the viral infection efficiency. When the OD_600_ was 0.6, the infection efficiency was significantly increased. However, a significant decrease in infection efficiency occurred when the OD_600_ exceeded 0.6 (**Figure 3A**). On the other hand, as OD_600_ increased from 0.3 to 0.6, chlorophyll content decreased significantly (**Figure 3B**). Although the data showed a significant decrease in chlorophyll content when the OD_600_ was greater than 0.6 (**Figure 3B**), the ulceration phenotype of some of the leaves was observed. Therefore, we inoculated the leaves using three different OD_600_ levels to determine the agrobacterium density that caused necrosis of leaf tissues (**Figure 3C**).

For resuspension solutions with OD_600_ > 0.8, the leaves showed necrotic spots and rapidly wilted and died (**Figure 3C**). Therefore, although the infection rate increased with increasing OD_600_ values, considering the activity of the practical factors, we used an OD_600_ of 0.6 as the optimum concentration for the *K. obovata* VIGS infection system.

### RNAi transient assay of *KoPDS* gene using the established VIGS system

**Figure 4:**
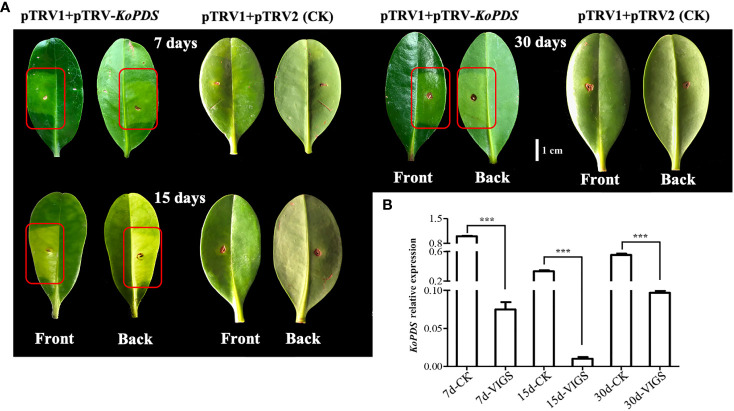
*KoPDS* silencing in *Kandelia obovata* and *KoPDS* relative expression after infection. **(A)** Silencing of *KoPDS* in *K. obovata* leaves by pTRV2-*KoPDS* (VIGS). The albino phenotype began to appear 7 days after infection, with the most severe effects seen at 15 days and some recovery apparent at 30 days. pTRV2-pTRV1 was used as control (CK). The red boxes indicate areas of infected leaves that appear to be fading green. **(B)** Comparison of relative *KoPDS* gene expression in control and VIGS plants at 7, 15, and 30 days after infection. *Koactin* was used to as an internal control. The 2^-△△Ct^ method was used to calculated expression data, which were replicated three times. Statistical analyses were performed using SPSS 17.0, and significance was assessed with Tukey’s Test at P < 0.05. All experiments included three biological replicates. Five leaves derived from five individual plants were collected for each biological replicate in each experiment. *** indicate that the data are directly significantly different (P < 0.01).

Silencing of the *PDS* gene, a key gene in carotenoid synthesis, can promote chlorosis symptoms in plants. In this study, chlorosis spots on the leaves of *K. obovata* mangrove plants appeared approximately 7 days after infection (**Figure 4A**). We selected infected leaves for RT-qPCR quantification at 7, 15, and 30 days after infection. The gene silencing was effective and lasting, as evidenced by significant reductions in *PDS* gene expression of 92.5%, 95.6% and 85.6% on day 7, 15 and 30 after infection, respectively (**Figure 4B**). Together, these results show that the VIGS system established in this study can achieve more than 90% silencing efficiency at 7 days, with peak of silencing efficiency occurring around 15 days and maintenance of 80% effective gene silencing by 30 days post infection.

### RNA-seq analysis and identification of *KoCab* functions on *K. obovata* leaves

To investigate the silencing effect of the VIGS system on the target genes and whether it has additional effects on the leaves that interfere with the experimental results, we analyzed the effects of the pTRV1+pTRV2 system on normal leaves using comparative transcriptome analysis. RNA-seq analysis was performed on normal leaves, leaves infected with the pTRV1+pTRV2 vector, or leaves infected with pTRV1+pTRV-*KoCab*. After discarding low-quality reads and adaptor sequences, a total of 55.70 Gb clean data were obtained from these samples. The Clean Reads of each sample were sequenced against the designated reference genome separately, and the mapping ratio ranged between 96.38% and 97.13%. Correlation analysis showed that the biological duplicates of leaves from different treatments were highly correlated. A total of 2,323 putative new genes not present in the genome were identified, of which 1,421 genes were annotated by databases, including the NCBI Non-Redundant Protein Database (NR), Swiss-Prot Protein Database (Swiss-Prot), Gene Ontology (GO), Orthologous Groups Database (COG), EuKaryotic Ortholog Groups of proteins database (KOG), Protein Family Database (Pfam), and the Kyoto Encyclopedia of Genes and Genomes (KEGG). A total of 8,395 genes were identified to be differentially expressed by pairwise comparison. Among them, 3,008 and 3,890 genes were differentially expressed on leaves infected with the pTRV1+pTRV2 vector and pTRV1+pTRV-*KoCab*, respectively.

**Figure 5:**
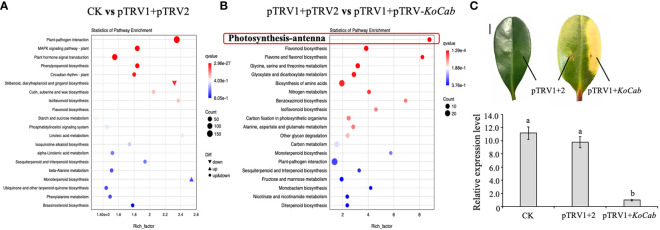
Transcriptome analysis of the effect of the VIGS silencing system on *K. obovata* leaves. **(A)** *K. obovata* leaves infected with pTRV1+pTRV2 for 15 days were used for transcriptome analysis, and normal growing leaves were used as controls (CK). **(B)** After the appearance of greening in the leaves (about 15 days of infection), the regions infected by pTRV1+pTRV2 and pTRV1+pTRV-*KoCab* were assessed separately for comparative transcriptome analysis. **(C)** Leaves was infected with different treatments and the phenotypic differences can be detected by 15 days after infection. Normal growing leaves were used as controls (CK). The regions infected with pTRV1+pTRV2 or pTRV1+pTRV-*KoCab* were labeled pTRV1 + 2 or pTRV1+*KoCab*, respectively. The scale indicates 1 cm. The relative expression of *KoCab* under different infection treatments is shown. *Koactin* was used to as an internal control. The 2^-△△Ct^ method was used to calculated expression data, which were replicated three times. Different letters indicate that the data are directly significantly different (P < 0.05).

According to the results of KEGG enrichment analysis and GO annotation, infection of the pTRV1+pTRV2 system resulted in a total of 411 differentially expressed genes that were assigned mainly to plant-pathogen interactions, the MAPK signaling pathway, phytohormone signaling, and the phenylalanine biosynthesis pathway (**Figure 5A**). Notably, on leaves infected with pTRV1+pTRV- *KoCab*, we not only saw a chlorophyll fading phenotype, but also found significant down-regulation of eight photosynthetic-antenna protein genes, together with a down-regulation of the flavonoid biosynthesis and flavone and flavonol biosynthesis pathways (**Figure 5B**). Net photosynthetic rate measurements showed that the difference between the infection with pTRV1+pTRV2 and the normal leaves was not significant, while the photosynthetic rate of the leaves infected with pTRV1+pTRV-*KoCab* was barely detectable after the appearance of the albino phenotype (data not shown).

On day 15 post-infection, a distinct greenish phenotype was observed and *KoCab* gene expression was significantly reduced by more than 95% (**Figure 5C**). These results again demonstrate that gene silencing is effective.

## Discussion

As one of the most carbon-rich ecosystems worldwide (Senger et al., 2021), mangroves are mostly found in tropical and subtropical coastal regions. Carbon sequestration by mangroves also plays a vital role in mitigating global warming and reducing greenhouse gas emissions (Gu et al., 2022). However, mangrove forests worldwide are declining at a rate of 1-2% per year due to continuous human disturbance (Goldberg et al., 2020). Therefore, conservation of mangroves is a matter of great urgency.

Photosynthesis is an indispensable part of mangrove survival and the carbon sink in special coastal habitats. PSII is the site where water is oxidized to oxygen during photosynthesis (Pagliano et al., 2013). The chlorophyll a-b binding protein is an important part of PSII, which is a class of proteins encoded by nuclear genes. In addition to light energy absorption and transfer, they play important roles in maintaining the structure of the cystoid membrane, regulating the distribution of excitation energy between the PSI and PSII photosystems, photoprotection, and adaptation to various environments. Therefore, studying the function of chlorophyll a-b binding protein in mangrove plants may be the key to resolving the regulatory mechanism of their photosynthetic carbon fixation. However, the identification of functional genes in mangrove plants has always been difficult, since few genetic transformation systems for mangroves have been developed. Establishment of the VIGS system for *Kandelia obovata* mangrove is therefore particularly important for accelerating research on functional mangrove genes.

Virus-induced gene silencing (VIGS) involves virus vectors to induce gene silencing in host plants. In recent years, VIGS has been used extensively in non-model plant species and, because of its simplicity, VIGS allows rapid phenotyping of the silenced genes while being virtually harmless to the plant from a genetic perspective. The success of the VIGS system is largely determined by the efficiency of infection and of silencing. Previous studies showed that infection efficiency is largely related to environmental conditions and infection method, whereas silencing efficiency is mostly related to the suitability of the virus vector used for the host plant (Deng et al., 2021). The TRV vector used in this study is able to infect most plants with few symptoms related to infection.

Susceptibility to TRV infection can vary from plant to plant or from plant to plant of the same species at different stages of growth (Deng et al., 2012; Naing et al., 2019). In this study, *K.obovasta* leaves exhibited a typical photobleaching phenotype, but leaves at different levels of development varied in their susceptibility to TRV. The 3rd-5th leaves from the top were selected as the best for this experiment because they had the highest silencing rate. In addition, we attempted to optimize the infection conditions by screening for pH and Agrobacterium density for more efficient VIGS use. The expression of virulence genes that are key for *Agrobacterium* infection was previously shown to be significantly increased with use of acidic virus resuspension buffer, and this increased expression translated to increased *Agrobacterium* infection rates (Baron et al., 2001). In the present study, we obtained similar results with the highest infection efficiency seen for pH 5.5-6.0, suggesting that *Agrobacterium* infection of mangrove plants is similar to that of other plants in some respects, but pH induces a degree of hypersensitivity in mangrove plants, such as delayed leaf greening, for reasons that require further investigation.

Although the silencing rate increased with the increase of Agrobacterium density, an Agrobacterium density that was too high (OD_600_>0.8) tended to cause ulceration of leaf tissues in *K. obovata*, while the intensity of photobleaching was highest at OD_600_ 0.6. Therefore, OD_600_ 0.6 was selected as the optimum density of Agrobacterium. In addition, the temperature of infection and cultivation also significantly affects *Agrobacterium* infection rates. Although higher temperatures favor spread of the pathogen after infection, the tumor-inducing activity of *Agrobacterium* rhizogenes is significantly inhibited above 29°C and may lead to a reduction in symptoms and duration of disease (Riker, 1926). In addition, the ability of *Agrobacterium* to form extracellular hairs (extracellular pilus, T-pilus) after infection of dicotyledonous plants is a major influence on infection rates. Temperatures >28°C significantly inhibit formation of T-pili and reduce the temperature stability of VIR proteins that further reduces the efficiency of *Agrobacterium* infection (Baron et al., 2001). Fullner and Nester (1996) showed that the optimal temperature for *Agrobacterium*-mediated transformation during infection and cultivation is 25°C, or as far below 28°C as possible. To activate the infection activity of *Agrobacterium*, the bacteria are resuspended in buffer before injection. Acetosyringone (AS) appears to be an essential element of this buffer, and mainly serves as a source of phenolic molecules that further promote expression of *vir* genes that promote *Agrobacterium* infection (Zou, 2011). Notably, although mangrove plant tissues contain high levels of phenolics and tannins, the concentration of AS in Agrobacterium-infected *K. obovata* was not different from that of other plants.

When the deletion of a gene shows significant morphological or color changes on the surface of the plant body, the use of this gene can quickly identify whether the VIGS system of the plant is established or not. In this study, based on the successful silencing of the phytoene desaturase gene, we also silenced the Cab gene and verified the function of this gene at the same time. The chlorophyll a-b binding protein has important effects on plant growth and development as well as physiological status. Knockdown of three genes, *Lhcb4*, *Lhcb5*, and *Lhcb6*, in *Arabidopsis thaliana* resulted in smaller, thinner leaves and fewer seeds (Chen et al., 2016; Chen et al., 2018). The results of the comparative transcriptome analysis showed that the *Cab* gene was successfully silenced under the VIGS system, but also triggered a reduction in the expression of some columns of *Cab* genes, together with the appearance of localized leaf bleaching. However, whether the leaf bleaching phenotype is caused by silencing of the target genes or is associated with down-regulation of multiple *Cab* genes will require further investigation. But it is certain that we confirmed that the target gene was silenced by qRT-PCR.

In this study, a VIGS system for *Kandelia obovata* mangrove plants was established for the first time that provides a good experimental system for study of functional genes in mangrove plants and provides new ideas and methods to further improve the silencing efficiency of the *Agrobacterium*-mediated VIGS system, which has high practical value.

## Conclusion

In this study, we constructed the first pTRV-*PDS* silencing vector for *Kandelia obovata* mangrove using tobacco rattle virus (TRV) and optimized infection conditions. The system was effective with a solution for infection that had OD_600_ 0.6 and pH 6.0. Under these conditions, the gene silencing efficiency could reach 95%.

## Data availability statement

The datasets presented in this study can be found in online repositories. The names of the repository/repositories and accession number(s) can be found below: CNCB Genome Sequence Archive, CRA011646.

## Author contributions

CP and JC conceived and designed the project. All authors collected samples and performed the experiment. MZ, YS, and CP analyzed the data. YS and YR wrote the manuscript. MZ and YR revised the manuscript. All authors contributed to the article and approved the submitted version.
