# Supplementary material for: Function verification of a chlorophyll a/b binding protein gene through a newly established tobacco rattle virus-induced gene silencing system in Kandelia obovata
Source: Front Plant Sci. 2023 Oct 3;14:1245555. doi: 10.3389/fpls.2023.1245555 (PMC10579580; doi:10.3389/fpls.2023.1245555)
Supplement: Supplementary file 1 [file DataSheet_1.pdf]

Supplementary data

Table S1. Primers used for pTRV vector construction and RT-qPCR.

**Primer**	Sequences
KoPDS-F	5'-cgtgattgaaggagatgcttatgt-3'
KoPDS-R	5'-gagtcagtgcgtgaaatcca-3'
KoPDS-OF	5'-taaggttaccgaattccgtgattgaaggagatgcttatgt-3'
KoPDS-OR	5'-cgcgtgagctcggtaccgagtcagtgcgtgaaatcca-3'
KoPDS-qF	5'-cgtgattgaaggagatgcttatg-3'
KoPDS-qR	5'-tgtgaacattgataacaggaactc-3'
Koactin-F	5'-accgaggctcctcttaatcc-3'
Koactin-R	5'-agctggcacattgaaggtct-3'
KoCab-F	5'-ggttacccaggtggactttg-3'
KoCab-R	5'-ccagtaccagtgtagatgtgttg-3'

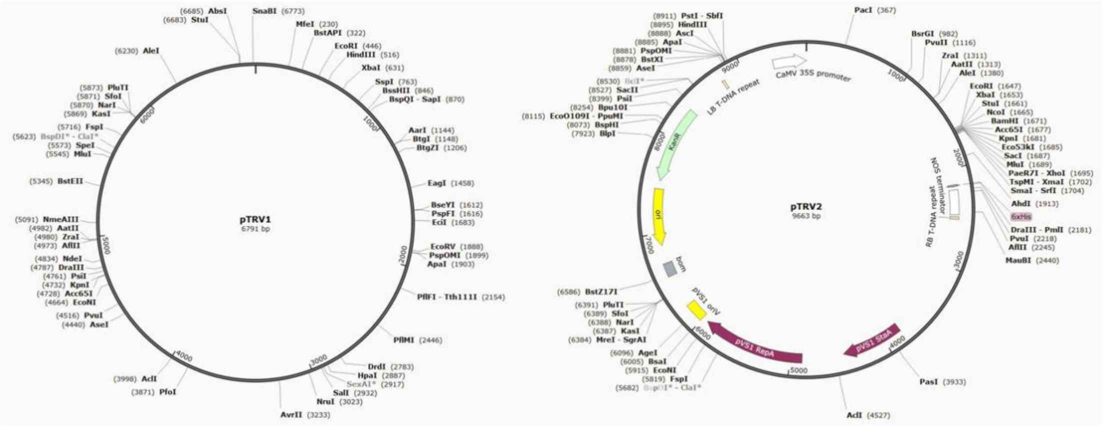

Figure S1. Schematic diagram of pTRV1 and pTRV2 construction.
